# Development of spectroelectrochemical microscopy for the real-time study of electrochemical surface processes

**DOI:** 10.1038/s41529-025-00728-x

**Published:** 2025-12-27

**Authors:** Matteo Olgiati, Markus Valtiner

**Affiliations:** Technische Universität Wien, Institute of Applied Physics, Wiedner Hauptstrasse 8-10, 1040 Wien, Austria

**Keywords:** Chemistry, Materials science

## Abstract

We present a multimodal methodology integrating time-resolved inductively coupled plasma mass spectrometry (ICP-MS) with operando reflected microscopy to characterise electrochemical surface processes. Enabled by a custom scanning flow cell, this approach allows simultaneous high-resolution optical inspection under controlled polarisation and continuous electrolyte flow. While ICP-MS and reflected microscopy have each advanced the study of electrocatalysis and corrosion, their direct combination correlates spatially resolved optical changes with quantitative, time-dependent dissolution kinetics. To illustrate its potential, we examined copper electrodes in dilute NaCl solutions with and without 2-mercaptobenzothiazole (2-MBT), a well-established corrosion inhibitor. The joint analysis distinguished mechanistic regimes of cathodic and oxide dissolution, uniform corrosion, passivation, and localised breakdown by linking morphology and optical features with dissolution profiles. Beyond this case, the methodology provides a versatile platform for *operando* electrochemical interface characterisation, bridging dynamic surface phenomena with kinetic reactivity.

## Introduction

Given the increasing demand for sustainable energy resources, electrochemistry is at the forefront of research, as it offers the possibility to convert chemical compounds (e.g. CO_2_) into more valuable energy carriers via charge-transfer reactions between the surface of an electrode and a liquid electrolyte^1^. To unravel the complexity of such reactions, detailed characterisation of the solid-liquid interface is necessary under realistic operational conditions^2–4^. For electrocatalysis, for example, this means that surface analysis should take place while catalytic reactions are ongoing (i.e., *in operando*). For corrosion studies, on the other hand, information on the degradation rate can be crucial to predict protection and prevention strategies.

Nevertheless, several difficulties can be encountered when characterising electrochemically-active surfaces. Firstly, traditional surface analytical techniques [e.g., X-ray photoemission spectroscopy (XPS), Auger emission spectroscopy (AES), Low-energy ion scattering (LEIS), Secondary ion mass spectrometry (SIMS), etc.] operate in ultra-high vacuum environments, which are very far from electrodes realistic working conditions (i.e., submerged in a liquid phase). Secondly, solid-liquid interfaces are buried and sometimes not always accessible by all types of probes, which either limits the range of suitable interface-sensitive techniques or requires to revise and/or adapt experimental set-ups to accommodate model surfaces. Thirdly, a combination of multiple techniques is sometimes necessary to obtain a complete understanding on the electrodes behaviour under electrochemical control. Lastly, electrochemical reactions can also be time- and/or potential-dependent, thus implying that better understanding of electrochemical reaction kinetics can derive from real-time surface analysis under polarisation control.

Therefore, a set of characterisation techniques that can relate the structural evolution of electrodes to the surface-related heterogeneities in their activity and/or stability is of great interest. This is true for the case of electrocatalysis, where even noble metals catalysts like Pt^5–7^, Au^8,9^ and Ru^10^ were shown to undergo dissolution and morphological reorganisation under operating conditions. Atomic-scale structural defects, like different facet orientation^11,12^, or morphological restructuring induced by adsorbents^13,14^ can impact the oxidation state^15^, selectivity of catalytic reactions^16,17^ and, ultimately, the overall catalytic performance.

Also in the case of corrosion, surface defects and microstructural features in engineering alloys can make them vulnerable to localised corrosion phenomena^18^. For example, “trenching” around intermetallic constituents is typical for high-strength aluminium alloys^19,20^, while stainless steels mostly show pitting nucleation near MnS inclusions^21–23^ and intergranular corrosion establishment at Cr-depleted grain boundaries^24^.

**Fig. 1:**
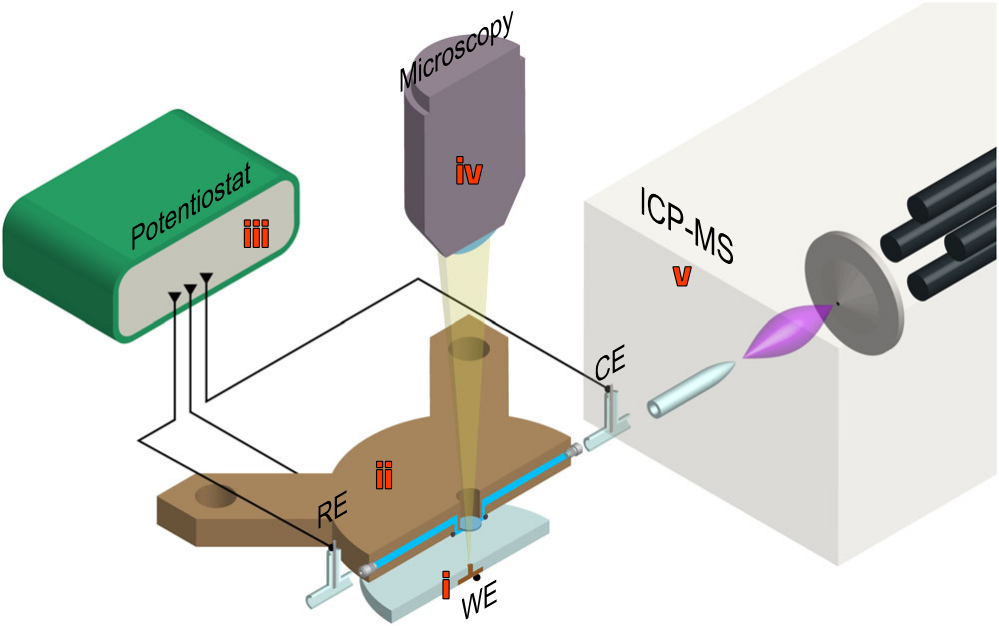
Experimental set-up schematics of the designed scanning-flow apparatus. The apparatus is designed to study the electrochemistry on micro-sized electrodes (**i**) inside a three-electrodes scanning-flow cell (**ii**) under polarisation (**iii**). The scanning flow cell enables simultaneous investigation of surface reflectance through optical microscopy (**iv**) and downstream analyte analysis with ICP-MS (**v**).

In this work, we enable the simultaneous coupling of downstream elemental analysis with inductively coupled plasma mass spectrometry (ICP-MS) and real-time reflected microscopy as an effective tool to study electrochemical surfaces in detail, in the attempt to bridge the electrode structural evolution under potential control with quantitative dissolution kinetics. Conceptually, this ”Spectroelectrochemical Microscopy” is illustrated in Fig. 1 (further details are provided in the Methods section).

A consistent number of works have already established the relevance of these techniques for the study of electrochemical systems. Time-resolved reflected microscopy, for example, was used to study the electrooxidation of polycrystalline Au^25^ and Fe^26^ electrodes, as well as localised degradation in stainless steel 316L^27^, high-strength aluminium alloys^28–30^ and formation of corrosion inhibitor layers^31,32^. Similarly, spectro-electrochemical techniques were formerly exploited to characterise several electrodes, e.g., the stability of Cu under catalytic reactions at different pH^33^, the mechanism of noble metal dissolution during oxygen evolution reactions^34^, metastable and stable pitting on stainless steel 316L^27^, crevice corrosion on Ni-based alloys^35^ and active-passive elemental dissolution kinetics of multi-principal element alloys^36^.

Yet, the simultaneous combination of spectro-electrochemistry with spatio-temporally resolved microscopy has not been explored so far, although it carries the potential to overcome some of the above-mentioned limitations, namely to relate (in-situ) the potential-dependent reactivity of electrodes to the evolution of surface structure and their stability in quantitative terms.

To validate the technique, we investigated the surface of Cu electrodes exposed to diluted solutions of NaCl, a well known corrosive medium, with presence or absence of dissolved 2-mercaptobenzothiazole (2-MBT). The interaction between Cu and 2-MBT serves as a well-known model system from previous experimental^37–39^ and theoretical^40–42^ investigations, which showed that 2-MBT acts as a corrosion inhibitor by adsorbing and binding to the surface of Cu. Our preliminary study shows that a combinatorial, real-time, and multi-scale approach like the one offered by the scanning flow cell can highlight characteristic qualitative features, as well as kinetic time-constants, that can aid the phenomenological interpretation of electrochemical surface reactions. In a simplified system like Cu exposed to 10 mM NaCl with or without addition of 2-MBT, we could make a clear distinction between different phenomena (namely, cathodic dissolution, oxide dissolution, uniform corrosion, passivation/corrosion inhibition and local pitting corrosion) by correlating reflected microscopy and online ICP-MS data to the applied potential. We show that analysis of surface reflectance, both spatially and temporally resolved, can facilitate the distinction of different surface phenomena, which can be further supported by the analysis of cumulative elemental dissolution kinetics.

## Results

### Correlative analysis of surface electrochemistry with real-time microscopy and mass spectrometry

**Fig. 2:**
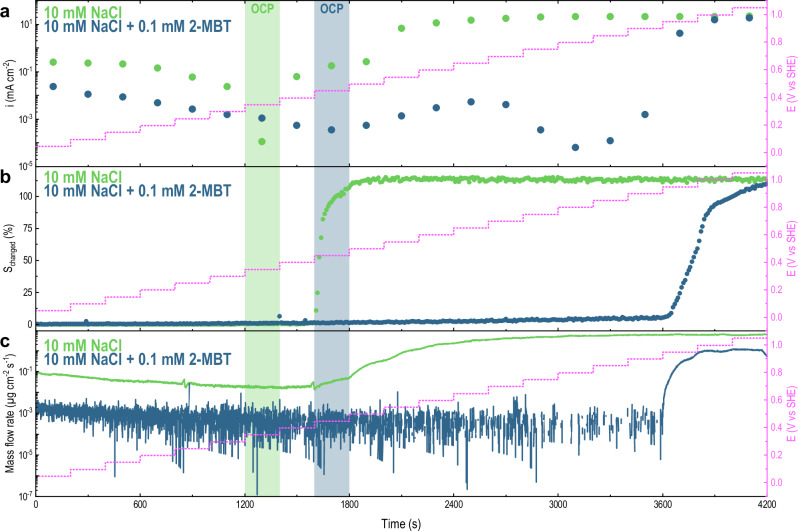
Time- and potential-dependent electrochemistry of Cu under polarisation conditions. The panels represent: (**a)** electron current density averaged for each potential step, (**b)** cumulative macroscopic optical response of the electrode’s surface calculated from Equation (1) (see Methods), (**c)** mass dissolution flow rate of Cu detected with ICP-MS.

Figure 2 summarises the characterisation of Cu electrodes in diluted NaCl in presence (blue) or absence (green) of 2-MBT. This was performed with an in situ scanning flow cell (shown in Fig. 1 and described in Methods), which allows for time- and potential-resolved characterisation, as shown by the potential (E) *vs* time magenta lines depicted in Fig. 2a–c.

More specifically, Fig. 2a shows the polarisation characteristics, reconstructed by averaging the current density at each potential step. Figure 2b shows the cumulated optical response on the electrode surface during immersion (*S*_changed_ %, see Eq. (1)). Figure 2c reports the Cu dissolution mass flow rate measured in real time at ICP-MS, while the corresponding current density (i_Cu_) is reported in Fig. S1.

The following observations can be summarised from Fig. 2:

*Immersion in 10* *mM NaCl*: In the selected potential window, both cathodic and anodic areas are scanned as indicated by the point of charge reversal denoted as ”OCP” (i.e., Open Circuit Potential) in Fig. 2a. Based on the current density response, three characteristic electrochemical regimes can be identified: (i) A cathodic polarisation regime (from 0.05 to 0.35 V vs SHE) is visible, denoted by a diffusion-limited current density, likely to be related to O_2_ reduction reactions. (ii) Just above the OCP (i.e., from 0.35 to 0.50 V *vs* SHE), Cu displays active anodic behaviour, denoted by a monotonically increasing current density as a function of potential (within the range of 10^−1^ mA cm^−2^). (iii) A final stage where Cu still displays active behaviour but with considerably higher current density (10^0^–10^1^ mA cm^−2^) becomes visible from 0.55 V until 1.05 V vs SHE.

Quantification of optical surface reactivity as a function of time and applied potential (Fig. 2b) allows to also identify these three characteristic stages as follows: (i) For potentials between 0.05 and 0.4 V vs SHE, no significant activity is observed as *S*_changed_ remains stable at 0%. (ii) At 0.45 V *vs* SHE the optical response starts to increase very rapidly until all of the exposed surface becomes affected (*S*_changed_ = 100%). (iii) At higher potentials (E > 0.45 V vs SHE), the *S*_changed_ curve saturates and stabilises at a constant value, although it becomes greater than 100%, indicating that an area larger than the electrode’s surface area is affected. This effect will be further discussed below.

From the Cu dissolution flow rate in Fig. 2c, two main regimes can be identified: (i) The first spans from 0.05 V vs SHE until 0.45 V vs SHE, covering the whole cathodic branch, the OCP and the first two anodic polarisation steps above OCP (0–1800 s). The mass flow rate remains overall within the range of 10^−2^ μg cm^−2^ s^−1^, despite the slope shows variations as a function of the applied potential. Namely, a decreasing dissolution rate is seen for more positive potentials in the cathodic branch. It seems to stabilise to a minimum at OCP and at 0.4 V vs SHE, and it starts increasing again at 0.45 V vs SHE. (ii) The second spans from 0.5 V vs SHE until the end of experiment (1800–4200 s) and displays a one-to-two orders of magnitude increased dissolution rate (10^−1^–10^0^μg cm^−2^ s^−1^).

*Immersion in 10* *mM NaCl* *+* *0.1* *mM 2-MBT*: Overall, a much lower current density was measured in presence of 2-MBT in solution (almost one order of magnitude), which could indicate a decreased surface activity. Four stages could be identified based on the polarisation curve: i) The first is characterised by cathodic polarisation from 0.05 V until 0.45 V vs SHE, with a seemingly diffusion-limited current density between 0.1 and 0.35 V vs SHE. The cathodic current density is one order of magnitude lower than in 10 mM NaCl. (ii) The second spans from 0.5 to 0.65 V vs SHE, where the anodic current density seems to increase, although remaining considerably lower than immersion in 10 mM NaCl (10^−3^–10^−2^ mA cm^−2^). (iii) The third spans from 0.65 to 0.9 V vs SHE, where the current density further decreases of two orders of magnitude (10^−4^ mA cm^−2^) in what looks like a passive region. The maximum of current density at 0.65 V vs SHE seems to denote an active-passive transition potential (i.e., the Flade potential). (iv) The fourth goes from 0.95 to 1.05 V vs SHE, where the current density increases by several orders of magnitude (10^0^–10^1^ mA cm^−2^), denoting the establishment of a transpassive region. The breakdown potential of 0.95 V vs SHE could therefore represent the critical pitting potential.

Three main phases are discernible in the optical response: (i) The first covers the cathodic polarisation from 0.05 V until 0.45 V *vs* SHE, where no significant variations in the cumulated optical response are visible (S_changed_ = 0%). (ii) The second period covers the active-passive region (approximately from 0.5 to 0.9 V vs SHE) and is characterised by moderate and slowly increasing activity, despite affecting limited areas of the exposed electrode (*S*_changed_ ~ 5–6%). (iii) The third period coincides with the onset of the transpassive region (from 0.95 to 1.05 V vs SHE) and is marked by a strong increase of the optical response that causes the whole surface area to be affected (*S*_changed_ = 100%).

The Cu dissolution profile in Fig. 2c seems to suggest three stages: (i) The first spans from 0.05 to 0.65 V vs SHE, covering the cathodic branch and a large portion of the anodic branch until the Flade potential (0.65 V vs SHE), where the mass flow rate remains overall in the range of 10^−4^μg cm^−2^ s^−1^. The noise in the curve compared to immersion in 10 mM NaCl is due to the vicinity of the signal to the limit of detection (≈ 0.1 ± 0.3 ppb). Despite this vicinity, a well discernible decreasing dissolution rate is visible for more positive potentials, which further stabilises at the OCP and above it. ii) The second stage spans between 0.65 V and 0.9 V vs SHE, where Cu dissolution remains low (~10^−5^–10^−4^μg cm^−2 ^s^−1^) and fluctuates around the limit of detection (occasionally below the limit of detection, as indicated by the missing data points in the line profile). (iii) The third stage coincides with the onset of transpassive polarisation at 0.95 V vs SHE, where enhanced Cu dissolution rate occurs (10^−1^–10^0^μg cm^−2^ s^−1^).

In summary, the results depicted in Fig. 2 show a good correlation between electrochemistry, the optical and dissolution activities, respectively. As expected, presence of 2-MBT in solution has a strong impact on the overall electrochemistry of Cu, since it lowers the surface activity and delays the onset of dissolution. For this reason, we analysed the electrode activity and kinetics more closely, in direct correlation with localised microscopic visualisation.

### Surface reactivity studied with reflectance analysis

**Fig. 3:**
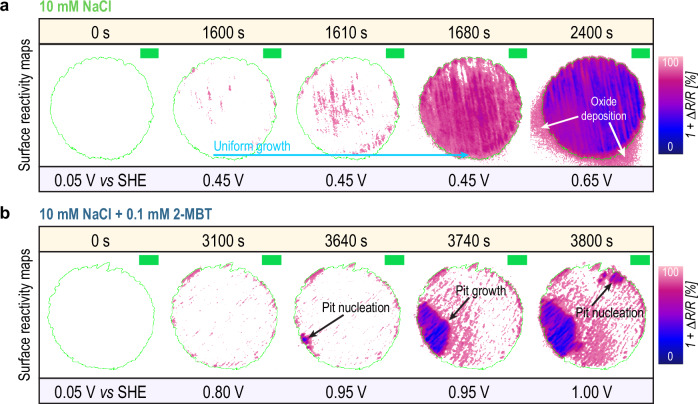
Surface reactivity maps of the electrode surface under immersion and under potentiostatic control based on the variation in surface reflectance over time. **a** refers to immersion in 10 mM NaCl while (**b**) refers to immersion in 10 mM NaCl + 0.1 mM 2-MBT. The circular green line indicates the position and perimeter of the micro-sized electrode surface. All the green scale bars in the maps represent 20 μm.

**Fig. 4:**
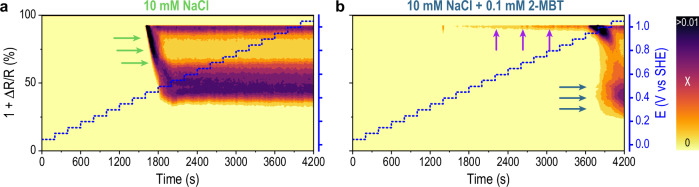
Time- and potential-dependent evolution of surface reflectance distribution under polarisation control. **a** refers to immersion in 10 mM NaCl, while (**b**) refers to immersion in 10 mM NaCl + 0.1 mM 2-MBT. The blue dashed line represents the time-variation of the applied potential.

Figure 3a, b report the locally resolved surface reactivity maps detected with in-situ reflected microscopy for immersion in 10 mM NaCl and 10 mM NaCl + 0.1 mM 2-MBT, respectively. Each map is calculated at a certain immersion time (or potential) and displays the relative change in reflectance at each time step (colour scale). The circular green line indicates the perimeter of the electrode’s surface (i.e., Cu phase is inside the circle, epoxy phase is outside the circle). The unprocessed micrographs (grey scale, 8-bit) are reported in Fig. S2. Figure 4, on the other hand, shows the time-evolution of the reflectance distribution for immersion in 10 mM NaCl (Fig. 4a) and 10 mM NaCl + 0.1 mM 2-MBT (Fig. 4b), respectively. The colour scale indicates the area fraction (*χ*) affected by a distinct change in reflectance (Δ*R*) at each time step.

The following observations can be made:

*Immersion in 10* *mM NaCl*: as previously observed in Fig. 2b, the onset of surface reactivity response takes place at 1600 s (0.45 V vs SHE). Only a few locations become electrochemically active, while the rest of the surface remains inactive. After 1610 s the nuclei become more sparse and uniformly distributed across the surface, whereas after 1680 s activity has propagated uniformly across the whole electrode area. This is consistent with the fast saturation observed in Fig. 2b (*S*_changed_ = 100%). The variation in reflectance seems to be similar and evenly distributed across the electrode surface. Indeed, a closer look at the time-evolution of reflectance (Fig. 4a) suggests that a high fraction of the surface seems to linearly decrease the reflectance value between 1600 and 1800 s (indicated by the green arrows), which could indicate that the entire exposed surface undergoes a similar phenomenological behaviour.

For higher anodic potentials (>0.65 V vs SHE), not only reflectance variations become more pronounced (1 + Δ*R*/*R* < 50%), but also some areas outside the perimeter of the electrode surface become affected (see white arrows in Fig. 3a), which explains the over-saturation observed in Fig. 2b (i.e., *S*_changed_ > 100%). Reflectance variations outside the perimeter of the electrode would suggest an activation of the epoxy phase, in which the electrode is embedded. Considering that the epoxy resin should not be sensitive to electrochemical potential variations, we instead attribute these changes in reflectance to a flow-assisted ejection and deposition of corrosion products on the surrounding epoxy phase. From Fig. 4a, a sharper decrease in reflectance can be observed already at 1800 s together with a broadening of *χ* over a larger range of 1 + Δ*R*/*R*. This trend seems to remain constant throughout the remaining immersion time.

*Immersion in 10* *mM NaCl* *+* *0.1* *mM 2-MBT*: as previously observed in Fig. 2b, the surface optical activation is seen just above the OCP and coincides with the establishment of an active-passive behaviour. Here (0.8 V vs SHE in Fig. 3b), surface reactivity consists of rather small clusters distributed across the exposed surface. This can also be seen in Fig. 4b, where an increasing *χ* with relatively high value of 1 + Δ*R*/*R* is observed in the active-passive region (see purple arrows). Some of the bigger clusters are more localised near the edges defining the electrode perimeter, where defects in the native oxide film are more likely. Given the non-monotonic trend between consecutive data points of *S*_changed_ in this region (see Fig. S3), the observed activity is likely to be related to reversible surface processes.

At the critical potential (0.95 V vs SHE, 3640 s) a more localised spot of intense reactivity nucleates (indicated with a black arrow in Fig. 3b). Due to the simultaneous increase of current density seen in Fig. 2a, this localised reactive cluster is likely to represent the nucleation of a stable pit. The nucleation event is also mirrored in the reflectance distribution displayed in Fig. 4b, where a rather fast development of *χ* with low 1 + Δ*R*/*R* (~30–40%) takes place at the critical pitting potential (see blue arrows). Such population is initially small (i.e., small *χ*), but it grows larger over time (larger *χ*). This is consistent with the surface reactivity maps in Fig. 3b, showing that the pit grows larger in size for longer immersion times (3740 s). For higher potentials (1.00 V vs SHE) nucleation of new pitting activity hot-spots can be observed at independent locations, as indicated by the black arrows in Fig. 3b.

### Electrode kinetics

**Fig. 5:**
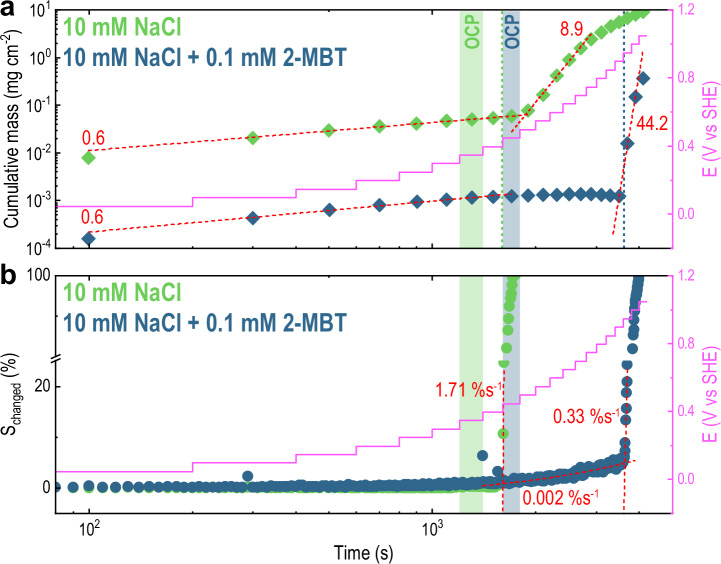
Electrode kinetics evaluated in terms of dissolution rate and optical response. **a** Cumulated dissolved mass of Cu during a linearly-stepped polarisation protocol for immersion in 10 mM NaCl (green) and 10 mM NaCl + 0.1 mM 2-MBT (blue). **b** Representation of *S*_changed_ (%) *vs* log(t) for immersion in 10 mM NaCl (green) and 10 mM NaCl + 0.1 mM 2-MBT (blue). The red dashed lines represent the slopes obtained from fitting, while the solid magenta lines show the variation of potential in time.

The time-resolved analysis provided by the scanning flow cell allows to gain better insights on the kinetics of electrode activity and stability. To be able to better identify and define different kinetic stages of corrosion, we calculated the cumulative Cu dissolution profile (reported in Fig. S4) by integrating the plots in Fig. 2c in time and averaging the result for each potential step. Due to the strong non-linearity of the curve, we re-plotted the cumulative mass in a logarithmic scale and compared to the *S*_changed_ curve (Fig. 5a and b, respectively). Representation on a log(t) plot enables identifying characteristic kinetic slopes that are depicted in red in Fig. 5.

The following observations can be made from Fig. 5:

*Cathodic Polarisation*: A slope of 0.6 characterises the Cu dissolution rate in the cathodic region for both immersion with (blue) and without (green) 2-MBT, even though the cumulative mass for immersion in 10 mM NaCl is almost two orders of magnitude larger than for 10 mM NaCl + 0.1 mM 2-MBT. This denotes that, even during cathodic polarisation, Cu dissolution can be detected with a similar characteristic time constant. As observed above, the optical response in this region is close to noise level (i.e., *S*_changed_ ~ 0%), suggesting that the Cu surface dissolution detected with mass spectrometry may take place at a scale that is within the shot noise of the microscope.

*Immersion in 10* *mM NaCl*: the cathodic slope of Cu dissolution (0.6) seems to extend for 100 mV further above the OCP (i.e., 0.45 V vs SHE). This also corresponds to the potential where we observe surface activation in Fig. 5b (the green dotted line in Fig. 5a represents the onset of activation). Such activation propagates fast on the surface (slope in *S*_changed_ of 1.71% s^−1^) and affects the whole surface area in less than 100 s. As also observed by reflectance analysis in Figs. 3a and 4a, surface reactivity is collectively and uniformly distributed across the electrode’s surface. Nevertheless, a transition to a faster dissolution kinetics only takes place at 150 mV above OCP (i.e., at 0.5 V vs SHE) and it is characterised by a slope of 8.9. Dissolution proceeds at this rate until 400 mV above the OCP, then it further slows down towards the end of the experiment.

The kinetic data presented in Fig. 5 suggest that the fast activation observed from reflected microscopy at 100 mV above OCP does not correspond with an equally fast dissolution of Cu. Conversely, Cu dissolution becomes more significant only at higher potentials (i.e., 150 mV above OCP). It is thereby reasonable to assume that the initial and fast variations in reflectance may not correspond to Cu corrosion (see Discussion).

*Immersion in 10* *mM NaCl* *+* *0.1* *mM 2-MBT*: Similarly to immersion in 10 mM NaCl, a cathodic slope of 0.6 appears. In contrast to immersion in 10 mM NaCl, the slope does not extend beyond OCP. Above OCP and throughout the passive region, the cumulated dissolved mass flattens into a plateau, meaning that no significant Cu dissolution is detected in this region or that the signal is below the detection limit. Minor changes in reflectance were still detected, as previously discussed for Figs. 2b, 3b and 4b. Their propagation rate is also rather slow (slope in *S*_changed_ of 0.002% s^−1^).

Only at the critical pitting potential (i.e., 0.95 V vs SHE) a new and fast dissolution kinetic regime appears (slope of 44.2), which corresponds to the nucleation and growth of local corrosion. Despite the increased dissolution rate compared to immersion in 10 mM NaCl, it is worth observing that the optical propagation of such localised activity is five times lower (0.33% s^−1^) compared to the one observed in 10 mM NaCl (1.71% s^−1^).

## Discussion

The combination of three simultaneous online techniques allowed us to characterise in depth the phenomenological behaviour of Cu exposed to diluted NaCl solution with and without addition of 2-MBT, without extensive use of ex-situ surface analytical techniques.

For immersion in 10 mM NaCl, the results outlined above showed non-negligible dissolution of Cu can already take place under cathodic polarisation, although this was not detected as a clear optical response. This indicates that the dissolution mechanism may involve thin layers below the resolution limit of the microscope. A proposed mechanism observed for Cu single crystals under cathodic polarisation involves the ejection of Cu adatoms to form 2D islands^43,44^, although this could not be directly attributed to dissolution. In our case, we infer that a similar mechanism may be present, and the flow of electrolyte on the surface may assist the detachment of these ejected 2D islands, thus resulting in detectable dissolution.

At higher (anodic) potentials just above OCP, a mismatch between a rapidly propagating optical activation and a rather slow Cu dissolution rate was observed. The latter, in particular, becomes faster only in the subsequent potential steps. By looking at the Pourbaix diagram (E *vs* pH) for Cu in chloride solutions at 25°C^45^, a transition from ${\,\mathrm{CuCl}\,}_{2}^{-}$ stability to Cu^2+^ stability is expected around 0.5 V vs SHE, which corresponds well to the potential where we observed both a jump in current density and an increased Cu dissolution rate. For this reason, we attribute the sustained optical activity detected before 0.5 V vs SHE to a uniform oxide dissolution assisted by Cl^−^ ions. For higher potentials, Porbaix diagrams predict Cu^2+^ to become thermodynamically stable^45^, suggesting that the dissolution of Cu^2+^ is thereby promoted. Our results seem to suggest that promoted dissolution of Cu^2+^ also corresponds to a transition to a higher-order time constant, representing an increased Cu dissolution kinetics. Preferential hot-spots of surface reactivity were not observed from reflectance maps, leading to the assumption that uniformly distributed corrosion takes place on Cu exposed to 10 mM NaCl.

As for immersion in 10 mM NaCl + 0.1 mM 2-MBT, the results presented above could reproduce the corrosion inhibitive behaviour of 2-MBT on Cu, as reported in several other works^37,46,47^. From the observations outlined above, we can conclude that Cu is protected by a chemisorbed film of 2-MBT that lowers its activity and limits its further dissolution. Presence of a 2-MBT inhibitive film can passivate the surface, thus increasing its stability at higher potentials. The generation of a passive-like region was also observed on other metal alloys^48^, which could indicate a similar inhibitive mechanism provided by 2-MBT.

Exposure to 2-MBT already at cathodic potentials shows a decreased activity of the surface. This is visible in the decreased current density in the cathodic branch of Fig. 2a compared to immersion in 10 mM NaCl. This could be a consequence of 2-MBT chemisorbing already at (low) cathodic potentials^49^, thus forming an inhibitive film on the surface. The Cu mass flow rate also suggests a suppressed dissolution in presence of 2-MBT. V. Garg et al.^47^ previously discussed the importance of pretreatments in the formation of the 2-MBT protective film, highlighting that 2-MBT can pre-adsorb on the native oxide, thus preventing its efficient cathodic reduction. This ”poisoning” effect will have dramatic consequences on the bonding mechanism of 2-MBT to the surface and the resulting protective properties^41^. Our results show that the Cu dissolution for cathodic polarisation has the same time constant as for immersion in 10 mM NaCl (same slope in Fig. 5a), but is quantitatively greatly suppressed. This could indicate that presence of 2-MBT may not change the cathodic dissolution mechanism, but it slows its kinetics down. For example, it was already shown that the mechanism of adatom ejection under cathodic potentials is affected by adsorbents that induce site-blocking effects^43^. Similarly, pre-adsorption of 2-MBT on the native oxide of Cu can induce site-blocking effects and substantially slow down the adatom ejection process.

During polarisation in the passive region, a gradually increasing optical response was observed in parallel to a stifled Cu dissolution. For this reason, we infer that the surface reactivity observed in the passive region may be related to a thickening of the 2-MBT inhibitive film. Previous characterisation with ToF-SIMS and XPS already proposed that 2-MBT can adsorb on Cu in multi-layers^47,50,51^. From the reflectance reactivity maps we were able to see that thickening is not uniformly distributed across the electrode’s surface, but preferential locations seem to be primarily affected. A possible explanation for this is the non-uniform reduction of the native oxide during cathodic polarisation, which may leave some unreduced oxide islands on the surface that get promptly passivated by 2-MBT. Moreover, preferential clusters of activity seem to be located near the edges of the electrode. A similar behaviour (i.e. activation starting at the periphery of the electrode, then propagating towards the centre) was also observed on Au^52^ and Fe^26^ electrodes. B. Niu et al.^52^ attributed this behaviour to a heterogeneous distribution of solution resistance across the electrode’s surface, which could enhance electron transfer in the peripheral regions of the electrode.

At a critical value of potential of 0.95 V vs SHE, the inhibitive film loses its stability locally and pit nucleation can be observed. From our results, pitting corrosion is characterised by a strong localised variation in surface reflectance with a sharp increase of the Cu dissolution rate. The dissolution rate for localised corrosion is much faster compared to uniform corrosion observed in 10 mM NaCl, albeit the optical propagation rate is comparatively slower (from Fig. 5b, 0.33% s^−1^vs 1.71% s^−1^) as less surface area is affected overall. Due to the localised nature of pitting corrosion, it is reasonable to assume that the optical propagation rate mostly represents the lateral growth of a pit, while resolution on its depth propagation may be limited by the microscope’s depth-of-field. Hence, the fast dissolution rate can be justified by an equally fast depth propagation.

In summary, we hereby demonstrate the capabilities of a novel spectro-electrochemical microscopy to characterise surface processes under electrochemical control. The flow cell can be easily incorporated in existing systems, providing a cheap and fast add-on to any spectro-electrochemical setup.

- The coupling of time-resolved mass spectrometry and reflected microscopy allows for the identification of distinctive phenomenological and kinetic hallmarks of Cu electrochemistry.
- Cathodic polarisation of Cu shows non-negligible dissolution, whose rate is slowed down by the adsorption of 2-MBT and formation of an inhibitive film.
- Exposure to NaCl under anodic polarisation showed that Cl^−^-assisted oxide dissolution takes place, before uniformly distributed corrosion initiates with an increased dissolution rate.
- 2-MBT adsorption already at low potentials does not eliminate cathodic dissolution of Cu. Dissolution of Cu is only significantly suppressed under anodic polarisation, where a passive-like region appears and thickening of the 2-MBT inhibitive film may take place. Only at a critical value of potential, the inhibitive film locally breaks down and localised corrosion (such as stable pitting) takes place, characterised by a faster dissolution rate than uniform corrosion.

## Methods

### Materials

All the chemicals in this research were used as-received and without further purification. Electrolytes solutions were obtained by dissolving NaCl (99%, Sigma Aldrich) in ultra-pure MilliQ water (resistivity of 18.2 M*Ω* cm^−1^) to a concentration of 10 mM. The concentration of 10 mM was chosen in compliance with the safety limits of ICP-MS. Further, corrosion inhibition properties were tested by dissolving 2-MBT (99%, Sigma Aldrich) into previously prepared 10 mM NaCl matrix solutions.

### Experimental set-up

We herby report the development of a new apparatus for the real-time investigation of electrochemical systems that combines electrochemistry, microscopy and mass spectrometry. The schematics of such experimental set-up is shown in Fig. 1 and the details are provided below. Experiments were repeated in duplicate to check on reproducibility (see Figs. S5–S7).

#### Sample preparation

We investigated the adsorption and desorption of thiazole-based inhibitors on pure Cu as a model system. For this purpose, Cu samples were prepared as micro-sized electrodes (see **i** in Fig. 1). This was achieved by embedding a Cu wire (99.99%, Goodfellow) in cold-curing epoxy resin discs, by keeping the wire longitudinal axis perpendicular to the axis of the epoxy disc. This allowed to expose only the cross section of the wire (nominal area ~0.785 mm^2^) to the electrolyte solutions. Before curing, the two-component resin mixture was degassed in vacuum for one hour. The Cu surface was further ground with SiC paper down to #P4000 grit size and polished with 1 *μ*m diamond suspensions. The samples were thoroughly rinsed and sonicated in ethanol and blow-dried in N_2_ before being transferred to the test cell.

#### Scanning flow cell

A dedicated scanning flow cell was designed to perform the investigation. This is represented schematically in **ii** of Fig. 1. The 3D-model was drawn in SolidEdge and then milled out of PEEK. The cell was designed to (i) sustain a continuous flow of electrolyte, (ii) perform electrochemistry in a three-electrodes configuration and (iii) allow for real-time optical inspection of the electrode’s surface under electrochemical control. For these reasons, the flow cell was equipped with the following:

- an o-ring (diameter 7 mm) that presses on the working electrode surface to guarantee leakage-free flow of electrolyte over the surface. It has to be noted that the diameter of the o-ring is much larger than the typical diameter of the electrode (~0.1 mm), thus implying that the o-ring presses on the epoxy phase and not directly on the electrode’s surface.
- T-connectors at the inlet/outlet of the flow cell body to host counter and reference electrodes, respectively.
- A sapphire optical window (Edmund Optics) to allow for in-situ microscopy of electrochemically-driven process.

#### Electrochemistry

Electrochemistry was carried out in a three-electrodes configuration and controlled with a PalmSens4 potentiostat (PalmSens, see **iii** in Fig. 1). Cu micro-electrodes were used as working electrodes and prepared as described above. A Pt wire was used as counter electrode, whereas an Ag wire was calibrated (see Fig. S8) and used as a pseudo-reference electrode. Pseudo-polarisation curves were reconstructed from linearly-stepped polarisation protocols. In particular, we used a potential profile that varied linearly between 0.05 and 1.05 V vs SHE in steps of 50 mV and 200 s duration. This was necessary in order to let mass spectrometry data equilibrate to steady-state at each potential step. The variation of potential *vs* time is shown in Fig. 2 as a red dashed line. Initial exposure at OCP was limited to 2–3 s thanks to the integrated real-time microscopy, which allowed to monitor the moving front of electrolyte in the flow cell. A preconditioning step of 90 s at 0.05 V vs SHE was also applied to allow for compensation of initial OCP transient dissolution, as well as for time-synchronisation of microscopy data.

#### In-situ reflected microscopy

High-resolution and time-resolved microscopy was obtained by placing the flow cell onto an optical breadboard with vibration dampening feet, where a commercial USB microscope (AF7515MZT4, DinoLite) was also installed. The flow cell was also clamped to a movable stage to facilitate the centring and focusing of the electrode surface in the microscope’s field-of-view and focus plane. The microscope was then positioned in front of the cell’s optical window (as schematically depicted in **iv** of Fig. 1) and frames were captured sequentially with a frequency of 0.1 Hz. Micrographs were also recorded with bright-field coaxial illumination and with a fixed value of exposure per second (lux s^−1^) in order to prevent under or over exposure during the measurement. The image resolution was ~0.3 μm per pixel.

#### ICP-MS

Downstream and time-resolved analysis of Cu dissolution was performed using inductively coupled plasma mass spectrometry (ICP-MS, Agilent 7900 ICP-MS, Agilent Technologies). This is schematically shown in **v** of Fig. 1. In short, the electrolyte exiting the scanning flow cell is nebulised into a mist that gets further ionised into an Ar plasma. Before the elements get filtered by a quadrupole mass filter and resolved by an electron multiplier detector, they pass through a collision cell saturated with He gas (flow rate of 5 mL min^−1^) to reduce polyatomic interference. Calibration of spectral line 63 corresponding to Cu isotope was performed prior to experiments using a multi-element standard containing 1 ppm of Cu (Agilent). The limits of detection for the two solutions used, i.e. 10 mM NaCl and 10 mM NaCl + 0.1 mM 2-MBT, were calculated to be 0.17 ± 0.04 ppb and 0.10 ± 0.38 ppb, respectively. Flow of the analyte was controlled with compressed air as reported elsewhere^35^ with an average flow rate of 5.4 ± 0.8 mg of solution per second. Spectral line of Yttrium isotope 89 was used as internal standard. Data were collected in time-resolved analysis mode and were time-synchronised with the starting time of the electrochemistry protocol. Delay time due to liquid flow in the capillaries (≈35 s) was calibrated with the method suggested elsewhere^53^ and compensated in the data displayed. Furthermore, the baseline relative to the limit of detection and matrix effects was subtracted from the data.

### Image analysis

The analysis of microscopy data was based on the same algorithms previously reported by S. Garcia et al.^29,30^ that can be executed with the open-source software ImageJ. In short, the protocol consists of:

- a first recursive repositioning based on rigid-body kinematics^54^;
- pixel-by-pixel evaluation of the change of reflectance (1 + Δ*R*/*R* %) over time^26^ together with a de-noising protocol based on the increase of the histogram’s lower threshold^30^;
- conversion of the reflectance changes into *surface reactivity maps* with a correlated colour scale expressing the value of 1 + Δ*R*/*R* %^30^;
- quantification of the rate of surface reactivity (*S*_changed_) based on Eq. (1)^29^, where N_TOT_ is the total number of pixels constituting the electrode’s surface and N(t) is the number of pixels with changed reflectance at each time step:
  1 $${S}_{changed}( \% )=\frac{N(t)}{{N}_{TOT}}\cdot 100.$$

Furthermore, time-dependent reflectance matrices were generated by sequential stacking of reflectance distributions (see Fig. 4 and Fig. S9 for explanation). The colour scale (i.e., *χ*) represents the area fraction in each image having a certain value of reflectance R_*i*_ or, in other words, a certain displacement Δ*R* from the initial reflectance value R_0_ (i.e., Δ*R* = *R*_*i*_ − *R*_0_). By defining N(R_*i*_) as the number of pixels with reflectance R_*i*_, *χ* can be expressed through Eq. (2) as:

2 $$\chi =\frac{N({R}_{i})}{{N}_{TOT}}.$$

## Supplementary information

Supplementary information

## Data Availability

The datasets generated and/or analysed during the current study are available in the TU Wien Research Data repository at 10.48436/twv06-83h42 (the link will be available upon acceptance).

## References

[1] Nitopi, S. et al. Progress and perspectives of electrochemical CO2 reduction on copper in aqueous electrolyte. *Chem. Rev.* **119**, 7610–7672 (2019). 31117420 10.1021/acs.chemrev.8b00705

[2] Chee, S. W., Lunkenbein, T., Schlögl, R. & Roldán Cuenya, B. Operando electron microscopy of catalysts: the missing cornerstone in heterogeneous catalysis research? *Chem. Rev.* **123**, 13374–13418 (2023). 37967448 10.1021/acs.chemrev.3c00352 PMC10722467

[3] Lin, Q. A primer to in-situ opto- and spectro-electrochemistry. *Curr. Opin. Electrochem.* **37**, 101201 (2023).

[4] Magnussen, O. M. et al. In situ and operando X-ray scattering methods in electrochemistry and electrocatalysis. *Chem. Rev.* **124**, 629–721 (2024). 38253355 10.1021/acs.chemrev.3c00331 PMC10870989

[5] Yanson, A. I. et al. Cathodic corrosion: a quick, clean, and versatile method for the synthesis of metallic nanoparticles. *Angew. Chem. Int. Ed.* **50**, 6346–6350 (2011). 10.1002/anie.201100471 PMC3166651 21626623

[6] Meier, J. C. et al. Degradation mechanisms of Pt/C fuel cell catalysts under simulated start–stop conditions. *ACS Catal.* **2**, 832–843 (2012).

[7] Topalov, A. A. et al. Dissolution of platinum: limits for the deployment of electrochemical energy conversion? *Angew. Chem. Int. Ed.* **51**, 12613–12615 (2012). 10.1002/anie.201207256 PMC3556695 23124819

[8] Cherevko, S., Topalov, A. A., Zeradjanin, A. R., Katsounaros, I. & Mayrhofer, K. J. J. Gold dissolution: towards understanding of noble metal corrosion. *RSC Adv.* **3**, 16516 (2013).

[9] Stumm, C. et al. Reduction of oxide layers on Au(111): The interplay between reduction rate, dissolution, and restructuring. *J. Phys. Chem. C.* **125**, 22698–22704 (2021).

[10] Frydendal, R. et al. Benchmarking the stability of oxygen evolution reaction catalysts: the importance of monitoring mass losses. *ChemElectroChem*. **1**, 2075–2081 (2014).

[11] Perez, J., Gonzalez, E. R. & Villullas, H. M. Hydrogen evolution reaction on gold single-crystal electrodes in acid solutions. *J. Phys. Chem. B* **102**, 10931–10935 (1998).

[12] Hori, Y., Takahashi, I., Koga, O. & Hoshi, N. Electrochemical reduction of carbon dioxide at various series of copper single crystal electrodes. *J. Mol. Catal. A: Chem.* **199**, 39–47 (2003).

[13] Amirbeigiarab, R. et al. Atomic-scale surface restructuring of copper electrodes under CO2 electroreduction conditions. *Nat. Catal.* **6**, 837–846 (2023).

[14] Behjati, S., Hajilo, M., Albers, M. & Koper, M. T. M. Anisotropic roughening of a Au(111) single-crystal electrode surface in HClO4 solution during oxidation–reduction cycles. *J. Phys. Chem. C.* **129**, 8915–8926 (2025). 10.1021/acs.jpcc.5c01177 PMC12086851 40395712

[15] Fuchs, T. et al. Anodic and cathodic platinum dissolution processes involve different oxide species. *Angew. Chem. Int. Ed.* **62**, e202304293 (2023). 10.1002/anie.202304293 37341165

[16] Huang, Y., Handoko, A. D., Hirunsit, P. & Yeo, B. S. Electrochemical reduction of CO2 using copper single-crystal surfaces: effects of CO* coverage on the selective formation of ethylene. *ACS Catal.* **7**, 1749–1756 (2017).

[17] Scholten, F., Nguyen, K. C., Bruce, J. P., Heyde, M. & Roldan Cuenya, B. Identifying structure–selectivity correlations in the electrochemical reduction of CO2: A comparison of well-ordered atomically clean and chemically etched copper single-crystal surfaces. *Angew. Chem. Int. Ed.* **60**, 19169–19175 (2021). 10.1002/anie.202103102 PMC8457179 34019726

[18] Lindell, D. & Pettersson, R. Crystallographic effects in corrosion of austenitic stainless steel 316L. *Mater. Corros.* **66**, 727–732 (2014).

[19] Kosari, A. et al. In-situ nanoscopic observations of dealloying-driven local corrosion from surface initiation to in-depth propagation. *Corros. Sci.* **177**, 108912 (2020).

[20] Boag, A., Hughes, A., Glenn, A., Muster, T. & McCulloch, D. Corrosion of AA2024-T3 part I: localised corrosion of isolated IM particles. *Corros. Sci.* **53**, 17–26 (2011).

[21] Muto, I., Ito, D. & Hara, N. Microelectrochemical investigation on pit initiation at sulfide and oxide inclusions in type 304 stainless steel. *J. Electrochem. Soc.* **156**, C55 (2009).

[22] Chiba, A., Muto, I., Sugawara, Y. & Hara, N. Pit initiation mechanism at MnS inclusions in stainless steel: synergistic effect of elemental sulfur and chloride ions. *J. Electrochem. Soc.* **160**, C511–C520 (2013).

[23] Li, D., Huang, F., Lei, X. & Jin, Y. Localized corrosion of 304 stainless steel triggered by embedded MnS. *Corros. Sci.* **211**, 110860 (2023).

[24] Ida, N., Nishimoto, M., Muto, I. & Sugawara, Y. Role of MnS in the intergranular corrosion and depassivation of sensitized type 304 stainless steel. *npj Mater. Degrad.* **8**, 2 (2024).

[25] Pfaff, S. et al. Operando reflectance microscopy on polycrystalline surfaces in thermal catalysis, electrocatalysis, and corrosion. *ACS Appl. Mater. Interfaces* **13**, 19530–19540 (2021). 33870682 10.1021/acsami.1c04961 PMC8288973

[26] Makogon, A., Noël, J.-M., Kanoufi, F. & Shkirskiy, V. Deciphering the interplay between local and global dynamics of anodic metal oxidation. *Anal. Chem.* **96**, 1129–1137 (2024). 38197168 10.1021/acs.analchem.3c04160

[27] Choudhary, S., Kelly, R. & Birbilis, N. On the origin of passive film breakdown and metastable pitting for stainless steel 316L. *Corros. Sci.* **230**, 111911 (2024).

[28] Godeffroy, L., Makogon, A., Gam Derouich, S., Kanoufi, F. & Shkirskiy, V. Imaging and quantifying the chemical communication between single particles in metal alloys. *Anal. Chem.* **95**, 9999–10007 (2023). 37327768 10.1021/acs.analchem.3c01258

[29] Denissen, P. J., Homborg, A. M. & Garcia, S. J. Interpreting electrochemical noise and monitoring local corrosion by means of highly resolved spatiotemporal real-time optics. *J. Electrochem. Soc.* **166**, C3275–C3283 (2019).

[30] Olgiati, M., Denissen, P. J. & Garcia, S. J. When all intermetallics dealloy in AA2024-T3: quantifying early stage intermetallic corrosion kinetics under immersion. *Corros. Sci.* **192**, 109836 (2021).

[31] Denissen, P. J. & Garcia, S. J. Reducing subjectivity in EIS interpretation of corrosion and corrosion inhibition processes by in-situ optical analysis. *Electrochim. Acta*. **293**, 514–524 (2019).

[32] Mopon, M., Mol, A. & Garcia, S. J. Effect of delayed inhibitor supply on AA2024-T3 intermetallic activity: a local in situ analysis with reflected microscopy. *Corros. Sci.* **230**, 111910 (2024).

[33] Speck, F. D. & Cherevko, S. Electrochemical copper dissolution: a benchmark for stable CO2 reduction on copper electrocatalysts. *Electrochem. Commun.* **115**, 106739 (2020).

[34] Cherevko, S. et al. Dissolution of noble metals during oxygen evolution in acidic media. *ChemCatChem*. **6**, 2219–2223 (2014).

[35] Dworschak, D. et al. Comparison of elemental resolved non-confined and restricted electrochemical degradation of nickel base alloys. *Corros. Sci.* **190**, 109629 (2021).

[36] Han, J. et al. Elementally resolved dissolution kinetics of a Ni-Fe-Cr-Mn-Co multi-principal element alloy in sulfuric acid using AESEC-EIS. *J. Electrochem. Soc.* **169**, 081507 (2022).

[37] Sharma, S. B., Maurice, V., Klein, L. H. & Marcus, P. Local inhibition by 2-mercaptobenzothiazole of early stage intergranular corrosion of copper. *J. Electrochem. Soc.* **167**, 161504 (2020).

[38] Sharma, S. B., Maurice, V., Klein, L. H. & Marcus, P. In situ scanning tunneling microscopy study of 2-mercaptobenzimidazole local inhibition effects on copper corrosion at grain boundary surface terminations. *Electrochim. Acta*. **378**, 138150 (2021).

[39] Wu, X., Wiame, F., Maurice, V. & Marcus, P. Moiré structure of the 2-mercaptobenzothiazole corrosion inhibitor adsorbed on a (111)-oriented copper surface. *J. Phys. Chem. C.* **124**, 15995–16001 (2020). 10.1021/acs.jpcc.0c04083 PMC7385852 32742539

[40] Chiter, F., Costa, D., Maurice, V. & Marcus, P. Dft investigation of 2-mercaptobenzothiazole adsorption on model oxidized copper surfaces and relationship with corrosion inhibition. *Appl. Surf. Sci.* **537**, 147802 (2021).

[41] Chiter, F., Costa, D., Maurice, V. & Marcus, P. Corrosion inhibition of locally de-passivated surfaces by DFT study of 2-mercaptobenzothiazole on copper. *npj Mater. Degrad.* **5**, 52 (2021).

[42] Chiter, F., Costa, D., Maurice, V. & Marcus, P. Atomic scale insight into corrosion inhibition: DFT study of 2-mercaptobenzimidazole on locally de-passivated copper surfaces. *J. Electrochem. Soc.* **168**, 121507 (2021).

[43] Kunze, J., Maurice, V., Klein, L. H., Strehblow, H.-H. & Marcus, P. In situ STM study of the effect of chlorides on the initial stages of anodic oxidation of Cu(111) in alkaline solutions. *Electrochim. Acta*. **48**, 1157–1167 (2003).

[44] Auer, A. et al. Self-activation of copper electrodes during CO electro-oxidation in alkaline electrolyte. *Nat. Catal.* **3**, 797–803 (2020).

[45] Alfantazi, A., Ahmed, T. & Tromans, D. Corrosion behavior of copper alloys in chloride media. *Mater. Des.* **30**, 2425–2430 (2009).

[46] Balaskas, A. C., Curioni, M. & Thompson, G. E. Effectiveness of 2-mercaptobenzothiazole, 8-hydroxyquinoline and benzotriazole as corrosion inhibitors on AA 2024-T3 assessed by electrochemical methods. *Surf. Interface Anal.* **47**, 1029–1039 (2015).

[47] Garg, V. et al. Enhanced corrosion inhibition of copper in acidic environment by cathodic control of interface formation with 2-mercaptobenzothiazole. *Electrochim. Acta*. **447**, 142162 (2023).

[48] Visser, P., Terryn, H. & Mol, J. On the importance of irreversibility of corrosion inhibitors for active coating protection of AA2024-T3. *Corros. Sci.* **140**, 272–285 (2018).

[49] Woods, R., Hope, G. & Watling, K. A SERS spectroelectrochemical investigation of the interaction of 2-mercaptobenzothiazole with copper, silver and gold surfaces. *J. Appl. Electrochem.* **30**, 1209–1222 (2000).

[50] Garg, V. et al. Inhibition of the initial stages of corrosion by 2-mercaptobenzothiazole adsorption and the effects of interfacial oxides on copper in neutral chloride conditions. *Corros. Sci.* **225**, 111596 (2023).

[51] Garg, V. et al. Adsorption of 2-mercaptobenzothiazole organic inhibitor and its effects on copper anodic oxidation in alkaline environment. *J. Electrochem. Soc.* **170**, 071502 (2023).

[52] Niu, B., Xie, R.-C., Ren, B., Long, Y.-T. & Wang, W. Radially distributed charging time constants at an electrode-solution interface. *Nat. Commun.* **15**, 5633 (2024). 10.1038/s41467-024-50028-2 PMC11224254 38965237

[53] Shkirskiy, V., Maciel, P., Deconinck, J. & Ogle, K. On the time resolution of the atomic emission spectroelectrochemistry method. *J. Electrochem. Soc.* **163**, C37–C44 (2015).

[54] Thevenaz, P., Ruttimann, U. & Unser, M. A pyramid approach to subpixel registration based on intensity. *IEEE Trans. Image Process.* **7**, 27–41 (1998). 18267377 10.1109/83.650848

