# Supplementary material for: Development of spectroelectrochemical microscopy for the real-time study of electrochemical surface processes
Source: Npj Mater Degrad. 2025 Dec 27;10(1):15. doi: 10.1038/s41529-025-00728-x (PMC12855015; doi:10.1038/s41529-025-00728-x)
Supplement: Supplementary file 1 — Supplementary information [file 41529_2025_728_MOESM1_ESM.pdf]

Supplementary Materials for
Development of Spectroelectrochemical Microscopy for the Real-Time Study of Electrochemical Surface Processes

Matteo Olgiati[1] and Markus Valtiner[1,*]

[1]Technische Universität Wien, Institute for Applied Physics, Wiedner Hauptstraße 8-10, 1040 Wien, Austria

*Corresponding author. Email: markus.valtiner@tuwien.ac.at

This PDF file includes:

Figures S1 to S9

Supplementary Figures

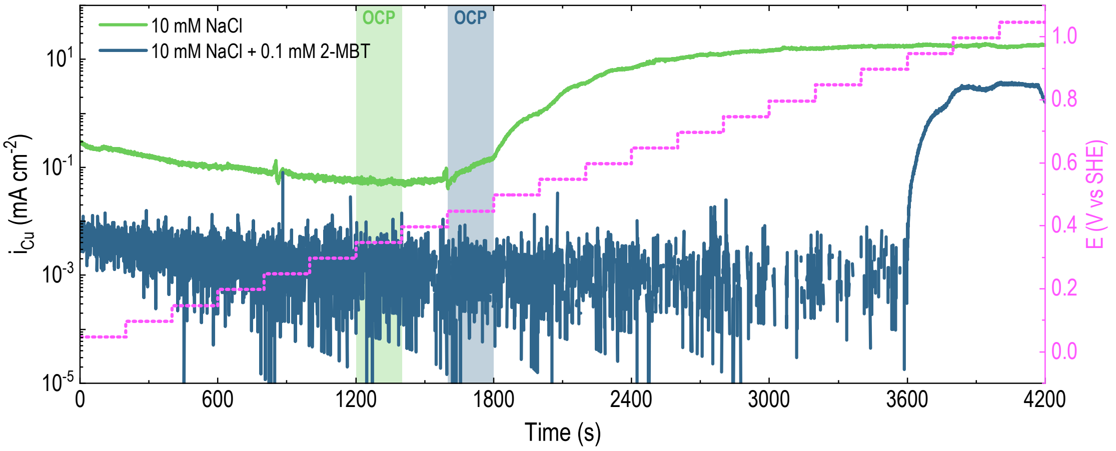

Figure S1: Calculated Cu current density (i_{Cu}) from mass dissolution profiles detected in ICP-MS. The green line corresponds to immersion in 10 mM NaCl, while the blue line corresponds to immersion in 10 mM NaCl + 0.1 mM 2-MBT. The dotted magenta line shows the applied potential as a function of time.

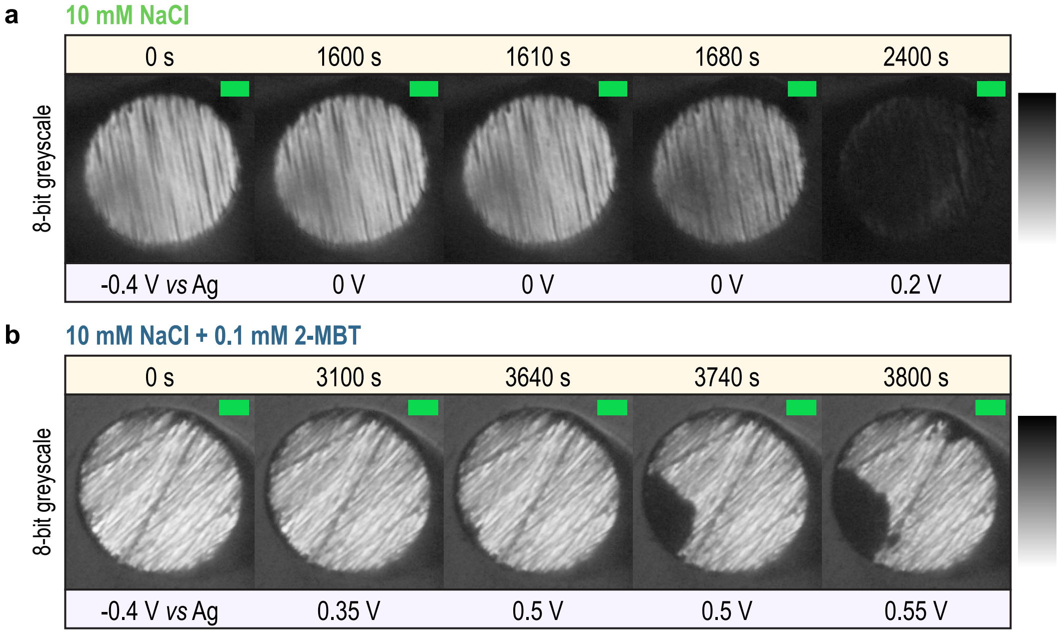

Figure S2: Optical micrographs (8-bit, greyscale) of the electrode surface under immersion and under potentiostatic control. Panel **(a)** refers to immersion in 10 mM NaCl while panel **(b)** refers to immersion in 10 mM NaCl + 0.1 mM 2-MBT. All the scale bars represent 20 μm.

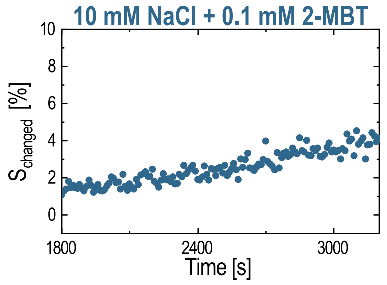

Figure S3: Close-up on the cumulated optical activity during immersion in 10 mM NaCl + 0.1 mM 2-MBT. The immersion time corresponds to polarisation in the passivity region.

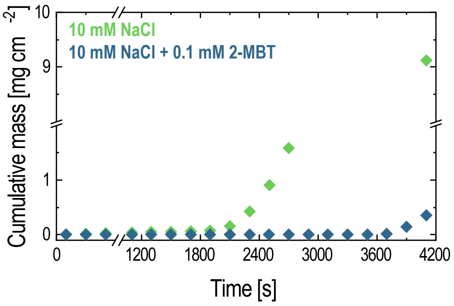

Figure S4: Trends of the cumulated Cu mass dissolution over time for immersion in 10 mM NaCl (green) and 10 mM NaCl + 0.1 mM 2-MBT (blue).

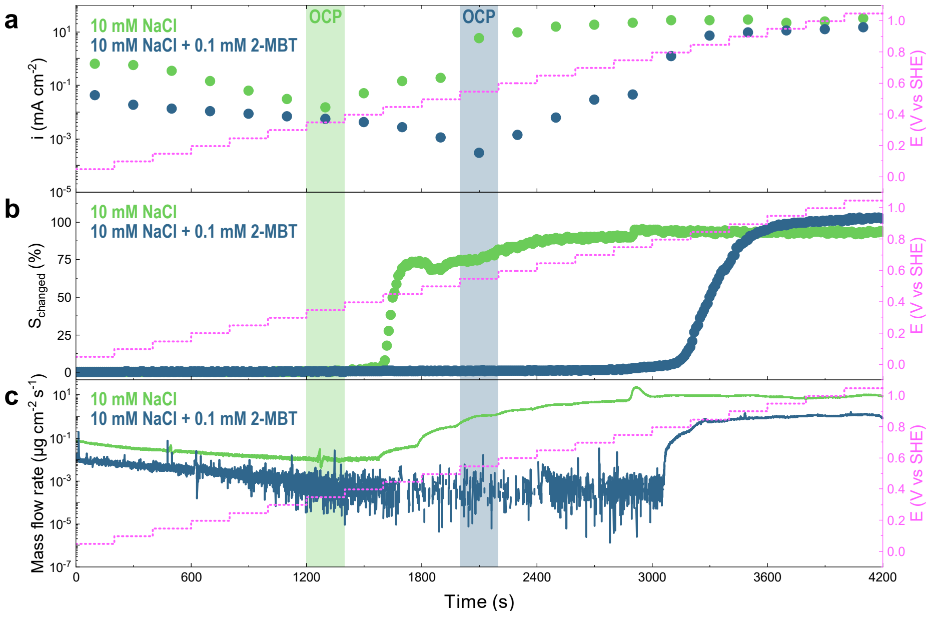

Figure S5: Reproducibility test for time- and potential-dependent electrochemistry of Cu under polarisation conditions. The panels represent: **(a)** electron current density averaged for each potential step, **(b)** cumulative macroscopic activity on the electrode's surface calculated by equation 1, **(c)** mass dissolution profile of Cu detected with ICP-MS.

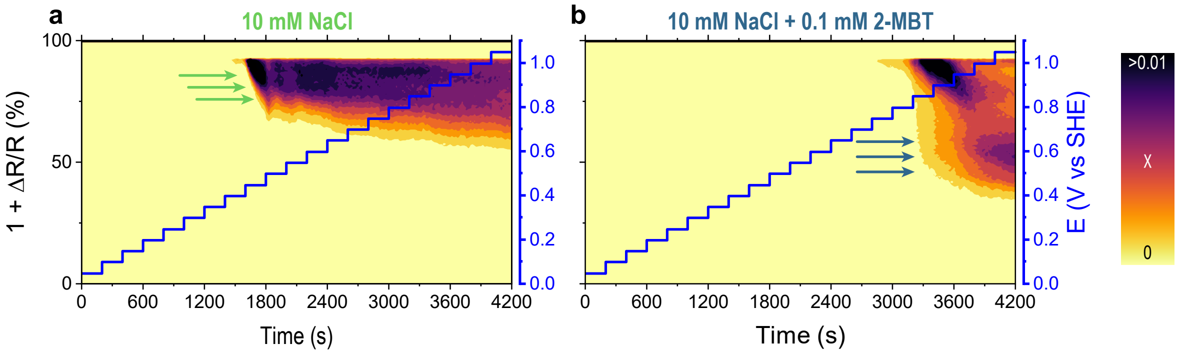

Figure S6: Reproducibility test of the time-evolution of surface reflectance distribution under polarisation control. Panel **(a)** refers to immersion in 10 mM NaCl, while panel **(b)** refers to immersion in 10 mM NaCl + 0.1 mM 2-MBT.

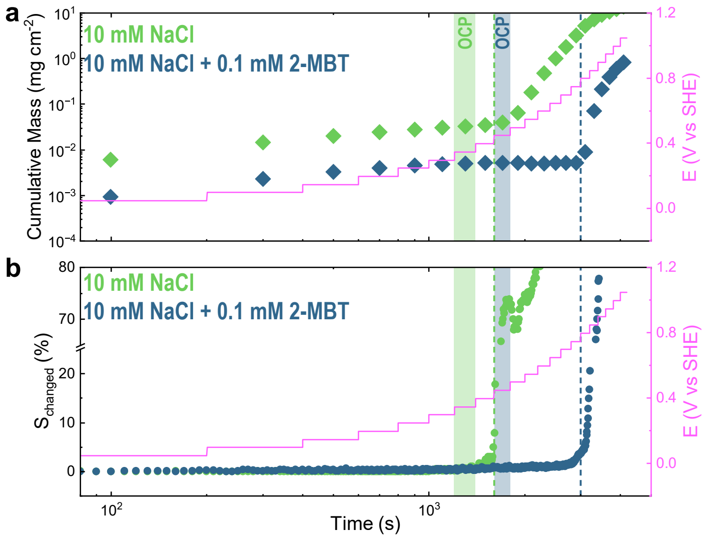

Figure S7: Reproducibility test for electrode kinetics evaluated in terms of dissolution rate and optical activity. **(a)** Cumulated dissolved mass of Cu during linear polaristion for immersion in 10 mM NaCl (green) and 10 mM NaCl + 0.1 mM 2-MBT (blue). **(b)** Representation of $S_{changed}$ (%) *vs* log(t) for immersion in 10 mM NaCl (green) and 10 mM NaCl + 0.1 mM 2-MBT (blue). The vertical dashed lines indicate the onset of fast optical activity.

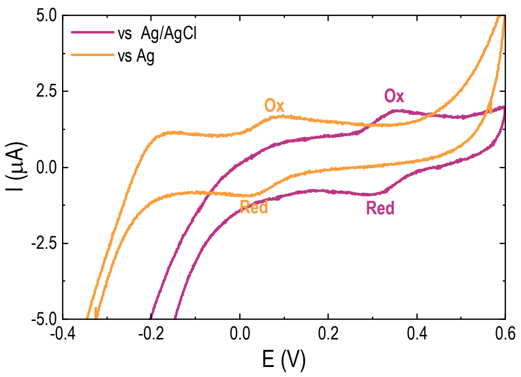

Figure S8: Cyclic voltammograms on Au in a solution of 0.1 M $NaClO_4$ saturated with ferrocene carboxylic oxide. The redox couple of ferrocene was monitored to evaluate the potential shift of a Ag electrode compared to a Ag/AgCl sat. KCl electrode. This calibration furtehr allowed to express the measured potentials *vs* Ag into a standard hydrogen electrode (SHE) scale.

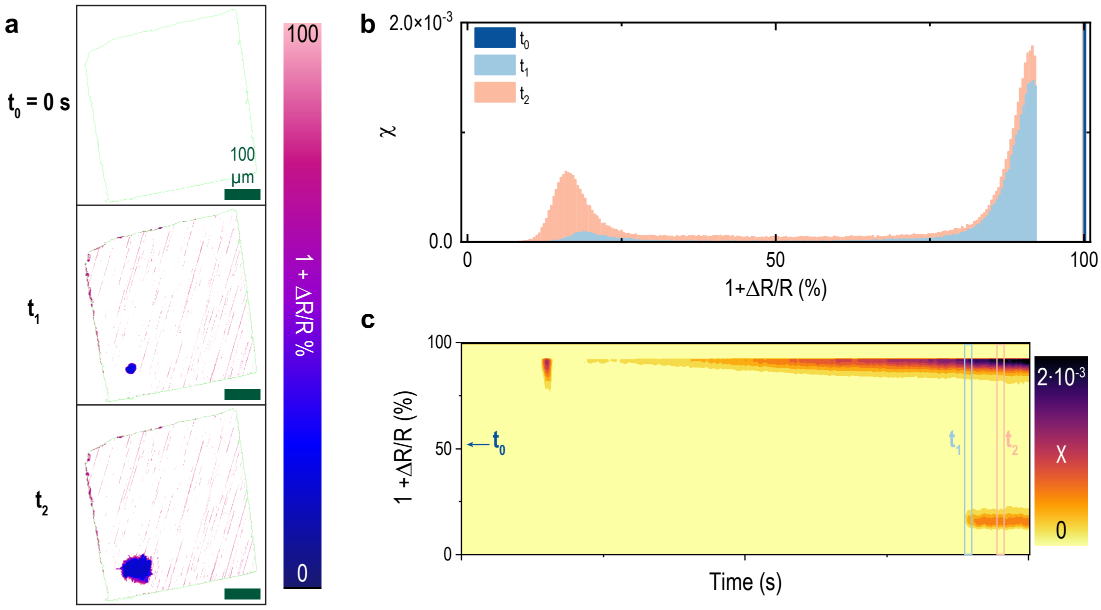

Figure S9: Process for the generation of reflectance matrices. **(a)** Activity maps of the electrode surface taken at different immersion times, namely t_0, t_1 and t_2 (t_0 = 0 s, $t_2 > t_1 > t_0$). The maps show different degrees of activity and, thereby, reflectance, as indicated by the colour scale. **(b)** For each activity map in (a), the reflectance distribution can be plotted to highlight the frequency (χ) of certain reflectance values. **(c)** The distributions from (b) can be stacked sequentially as a function of time and χ is converted to a colour scale in order to display time-dependent variations of reflectance.
